# Exercise Prescription Improve the Rehabilitation of a Child With Viral Encephalitis Sequelae: A Case Report

**DOI:** 10.3389/fped.2022.828014

**Published:** 2022-05-31

**Authors:** Yang Wang, Xiaodong Kang, Jiao Jiao, Jihe Zhou, Bik-Chu Chow, Julien S. Baker, Li Zhao, Siyu Liu

**Affiliations:** ^1^School of Sports Medicine and Health, Chengdu Sport University, Chengdu, China; ^2^Department of Child Rehabilitation, Sichuan Bayi Rehabilitation Center (Sichuan Province Rehabilitation Hospital), Chengdu, China; ^3^Dr. Stephen Hui Research Centre for Physical Recreation and Wellness, Hong Kong Baptist University, Kowloon Tong, Hong Kong SAR, China; ^4^Department of Sport, Physical Education and Health, Hong Kong Baptist University, Kowloon Tong, Hong Kong SAR, China; ^5^Faculty of Table Tennis, Badminton and Tennis, Chengdu Sport University, Chengdu, China

**Keywords:** child, viral encephalitis sequelae, exercise prescription, rehabilitation, brain activation, balance, gait

## Abstract

This study conducted a personalized exercise prescription intervention on a child with viral encephalitis sequelae (VES). The purpose was to observe the rehabilitation process from the aspects of brain activation, and the curative effects on balance function and gait. A further aim was to explore the possible nerve biomechanical mechanisms between the extent of brain activation and the improvement in balance function and gait. A 12-week exercise prescription was used as the treatment method, and functional near-infrared spectroscopy (fNIRS), balance function test system, plantar pressure distribution system, and 3D gait system were used to assess the effects of the rehabilitation process pre and post the intervention. Following the exercise prescription intervention: (1) fNIRS showed that brain activation in the S1–D1, S1–D2, S1–D3, S2–D1, S3–D2, S3–D3, S4–D3, S5–D5, S5–D6, S5–D7, S7–D6, S7–D7, S8–D7, and S8–D8 increased significantly (*P* < 0.05). (2) The balance test showed that the area of motion ellipse and movement length of the child with eyes open decreased significantly and area of motion ellipse, back and forth swing, left and right swing and movement length of the child with eyes closed all decreased significantly (*P* < 0.05). (3) The static plantar pressure distribution demonstrated that the pressure center of the left and right foot decreased significantly (*P* < 0.05) from 5.3° dislocation in a straight line in the sagittal plane to 1°; an increment of the pressure loading was found on the forefoot of both feet compared with what was recorded in the pre-test. (4) The testing results of the 3D gait system showed that she had a shortened time of unilateral support phase and prolonged swing phase on the affected leg (*P* < 0.05), compared to that of the non-affected leg. Furthermore, the dual support phase had also been prolonged (*P* < 0.05). Conclusion: 12 weeks’ individualized exercise training can enhance the activation in the motor areas and improve balance function and gait in a child with VES.

## Introduction

Viral encephalitis (VE) is a common disease in pediatrics, with an annual incidence of about 1.4 cases per 100,000 patients (1). It is estimated that there are about 150,000–300,000 cases of viral encephalitis reported every year (2). VE is caused by an inflammation of the brain parenchyma and is related to a variety of viral infections. There are more than 100 viruses that can cause infections of the central nervous system in the world (3). Almost 70% of viral encephalitis cases have no identified etiology (4), but may induce serious symptoms and long-term effects termed sequelae. In the acute stage of VE, the virus directly invades the central nervous system, which may lead to neuronal damage, and neurological tissues either disappear quickly or subside slowly over a period of time depending on the virus type and treatment. In the later stages of VE, the body reacts with immune system responses to the pathogens located in the central nervous system. Some children with VE are left with varying degrees of sequelae in the brain, one of which manifests itself as joint spasm, deformity, and even disability caused by diversified central motor disorders. The sequelae, along with mental stress, may further influence the patient’s quality of life. In turn, the most effective methods of alleviating the negative effects of these sequelae should be explored, to promote the rehabilitation of motor function and activate the brain’s nervous system.

As illustrated in a previous study (3), motor dysfunction of patients with viral encephalitis sequelae (VES) greatly limits their daily activities, which further exacerbates their decline in nerve-muscle function, including decreased muscle strength and motor ability. Thus, to restore the motor function of patients with VES, rehabilitation treatments should not only include medication, but also a comprehensive intervention program, such as a personalized exercise training program. Based on the neural plasticity theory, it is possible to restore motor function using designated exercise regimes. These mechanisms involve stimulation of the neurons located at various levels of the motor pathways, which can help adjust their excitability and obtain optimal motor output that can be controlled and well-coordinated. The restoration of motor function might be attributed to brain cortical reorganization following exercise training (5). Patients with VES have poor mobility of various joints, insufficient muscle strength and endurance, poor flexibility, and body asymmetry. Therefore, a single exercise session is not enough to meet the rehabilitation needs of these patients. Instead, an exercise prescription, which is a systematic and personalized function-training program, should be developed in conjunction with the patient’s health information. This information includes medical examination, exercise risk screening, and fitness test results. This should be able to meet the rehabilitative needs of patients with VES. The exercise prescription should include specified exercise parameters including Frequency (F), Intensity (I), Time (T), Type (T), Volume (V), and Progression (P). The exercise program is usually designed by a fitness instructor and/or rehabilitation specialist (6). As exercise prescription emphasizes personalized design for individuals of different ages, genders, physical conditions, and disease types, exercise can achieve the precision and status of “sports medicine” in helping certain individuals. In turn, if the exercise prescriptions are as effective as intended for the VSE patients, then it is considered that exercise is effective for rehabilitation purposes.

Patients with VES demonstrate motor dysfunction due to nervous system lesions. It is necessary to review VES rehabilitation, ideally through neuro-biomechanical assessment methods related to brain activation and motor function need to be used. Functional Near-infrared spectroscopy (fNIRS) is a non-invasive brain imaging technology that can detect cerebral cortical hemodynamic changes (7), assess the neural correlates of motor dysfunction, and consequently demonstrate the neural mechanisms underpinning intervention outcomes (8). In a previous study, fNIRS was used to detect sensitive changes in prefrontal cortex activation after hand training in children with unilateral cerebral palsy, indicating improved motor function (9). However, literature on the relationship between neuroimaging manifestations of encephalitis patients and recovery of motor function is very limited (10). Reviews of plantar pressure distribution and 3D gait testing showed that (11, 12), these assessment methods could effectively and objectively reflect the gait ability of patients with cerebral palsy and stroke (13, 14). However, these methods have not been used in the context of children suffering from VES. Existing studies mostly used the Berg Balance Scale (BBS) for balance and motor function of chronic stroke survivors (15), but quantitative assessments were seldom conducted. The above evaluation methods were used to assess the rehabilitation effects of cerebral palsy and stroke independently or in combination. It was noted that children with VES show similar symptoms of motor dysfunction as patients with cerebral palsy and stroke. Therefore, the neuro-biomechanical evaluation methods might also be feasible to effectively evaluate the rehabilitation progress of children with VES.

Given that the research lacks exercise prescriptions and evaluation methods applied to children with VES, this study aims (1) to evaluate the effectiveness of a personalized prescribed exercise program on the rehabilitation of a child with VES, from the aspects of brain activation, balance, and walking ability; (2) to explore the possible neuro-biomechanical mechanisms between brain activation and improvement of walking ability and balance function. Therefore, it was assumed that exercise prescription could improve the rehabilitation effects in a child with VES, and neuro-biomechanical assessments could monitor brain activity. This would also have the potential to produce a quantitative evaluation of the child’s changes in gait and balance ability.

## Case Report

An 8-year-old girl weighing 29.5 kg was diagnosed with VE at the age of 4 years and 5 months. According to the girl’s family, the patient had been to clinics at another five hospitals in different cities during the last 3.5 years (and therefore experienced the golden treatment period) before she came to this author’s clinic. In the first clinic, she could only walk on hand after the initial 2 months of physiotherapy and hydrotherapy. With another 1 year of physiotherapy, by adding repetitive transcranial magnetic stimulation in the second clinic, she was gradually able to walk independently. However, she still fell easily because of poor balance and motor function. During the following 2 years, she did not see noticeable progress to the point where she could retain basic motor function and walk up or down stairs with the assistance of a handrail, which she was able to do when receiving regular physical training. In periods where the training was intermittently suspended, her abilities would regress, and she was unable to jump or run until she participated in the current study. The present study was conducted in the Sichuan China Bayi Rehabilitation Hospital after obtaining ethical approval from the Research Ethics Committee of Sichuan China Bayi Rehabilitation Center (No. Ckzl-2020008). Informed consent was signed by the patient’s family after they were fully informed of the rehabilitation program, process, and related risks.

## Clinical Examination

After admission, the child underwent a neuro-musculoskeletal and motion-related functions assessment, which included:

(1) Range of Motion: Passive Range of Motion (PROM) (16) was utilized and scored “all normal”;
(2) Muscle strength: Manual muscle strength testing (MMT) (17) was performed, and the MRC scale of 0–5 from the UK Medical Research Council (18) was used as the grading method. Except for a score of 4 points for the bilateral carpal extensor muscle group, hip flexor muscle group, hip extensor muscle group, knee flexor muscle group, and ankle dorsiflexor muscle group, the remaining measures were graded 5;
(3) Spasm evaluation: The Modified Ashworth Scale (MAS) (19) showed that the grade of left plantar flexion was 2, right plantar flexion was 1+, and the remaining areas were graded 0;
(4) The evaluation results of the Gross Motor Function Measure (GMFM-88) (20) showed that the total percentage of GMFM-88 was 90.2%, and the gross motor function on the left side was weaker than that of the right side. The above evaluation results were later used as a reference for the development of the personalized exercise prescription program.

## Methods

### Intervention Method

In this study, the exercise prescription was designed according to The American College of Sports Medicine Guidelines (ACSM’s) and was formulated following the FITT-VP principle (6). The details of the exercise prescription program are listed as follows:

Frequency: The frequency of training was 5 days a week (from Monday to Friday), two training sessions per day (morning and afternoon session), thus a total of 10 training sessions per week;

Intensity: Intensity was determined by the maximum number of training repetitions, i.e.,: 1–6 RM was treated as high intensity, 7–12 RM as medium intensity, and 13–18 RM as low intensity (21, 22). All training was conducted using a combination of high and medium intensities;

Time: Training lasted for 12 weeks (23–25), with 1 h in the morning and afternoon training sessions;

Type: Training involved a combination of fast and slow movement, dynamic and static movement, command and demonstration activities, a combination of upper and lower limbs, complex movement and simple movement, resistance practice, and flexibility practice;

Volume: Gradual increases in the level of training complication and difficulty from Monday to Friday, with increases every month, using repetitions involving key motions and core muscle groups. Training intensity mainly increased in the last 3 weeks (week 10–12), while starting from week 6, unilateral resistance training was conducted with affected and non-affected sides and with additional training volume on the affected side;

Progression: The increments of training were adjusted according to recovery progress following training.

Notably, the child did not receive any other rehabilitation treatments for motor dysfunction during this period. Table 1 describes the weekly training content in detail.

**TABLE 1:** The main content of exercise prescription.

Time	Main content	RM
1st week	A combination of difficult- and easy-movement exercises; Exercises to correct flustered gait; Forefoot landing gait exercises; Learn the first section of broadcast gymnastics; Flexibility exercises.	16–18
2nd week	A combination of difficult and easy movement exercises; Resistance exercises to correct flustered gait; Correct the practice of inner eight characters; Forefoot landing gait exercises; Learn the second section of broadcast gymnastics; Flexibility exercises.	13–15
3rd week	Exercises to correct uncoordinated arm swings; Correct the resistance exercise of the inner eight; Forefoot resistance gait exercise; Core and lower limb resistance exercises with bare hands; Learn the third section of broadcast gymnastics; Flexibility exercises.	10–12
4th week	Correct uncoordinated resistance exercises with swinging arms; Correct the resistance exercise of the inner eight; An exercise in correcting overstep width; Full-body resistance exercises with bare hands; Learn the fourth section of broadcast gymnastics; Flexibility exercises.	10–12
5th week	Correct uncoordinated resistance exercises with swinging arms; Resistance exercises that correct step width too wide; Whole-body resistance exercises; Learn the fifth section of broadcast gymnastics; Flexibility exercises.	7–9
6th week	Correct uncoordinated resistance exercises with swinging arms; Resistance exercises that correct step width too wide; Whole-body resistance exercises; Balance exercises; Learn the sixth section of broadcast gymnastics; Flexibility exercises.	7–9
7th week	Comprehensive gait exercise; Balance exercises; Jumping over obstacles; Learn to run up and down stairs; Learn the seventh section of broadcast gymnastics; Flexibility exercises.	12–14
8th week	Comprehensive gait resistance exercise; Balance exercises; Sensitive exercise; Jumping over obstacles; Learn to spin up and down stairs; Learn to jump in place on both feet; Learn the eighth section of broadcast gymnastics; Flexibility exercises.	10–12
9th week	Comprehensive gait resistance exercise; Whole-body resistance exercises; Balance resistance exercises; Sensitive exercise; Learn to leap forward on your feet; Review the first and second sections of broadcast gymnastics; Flexibility exercises.	7–9
10th week	Comprehensive gait resistance exercise; Whole-body resistance exercises; Balance resistance exercises; Sensitive exercise; Learn to swing arm bipedal jump in place; Review the third and fourth sections of broadcast gymnastics; Flexibility exercises.	6–8
11th week	Comprehensive gait resistance exercise; Fast walking exercises with limited stride width and pace on the treadmill; Whole-body resistance exercises; Balance resistance exercises; Sensitive exercise; Learn to swing arms and jump forward on both feet; Review the fifth and sixth sections of broadcast gymnastics; Flexibility exercises.	4–6
12th week	Comprehensive gait resistance exercise; Variable speed walking exercises with limited step width and pace on the treadmill; Whole-body resistance exercises; Balance resistance exercises; Sensitive exercise; Different jumping combinations; Review the seventh and eighth sections of broadcast gymnastics; Flexibility exercises.	4–6

### Neuro-Biomechanical Assessment Methods

Neuro-biomechanical assessments were conducted prior to and following the 12-week exercise prescription intervention as “pre-test” and “post-test.” The neuro-biomechanical assessment methods included four main parts, described below.

#### Functional Near-Infrared Spectroscopy Technology Testing

**FIGURE 1:**
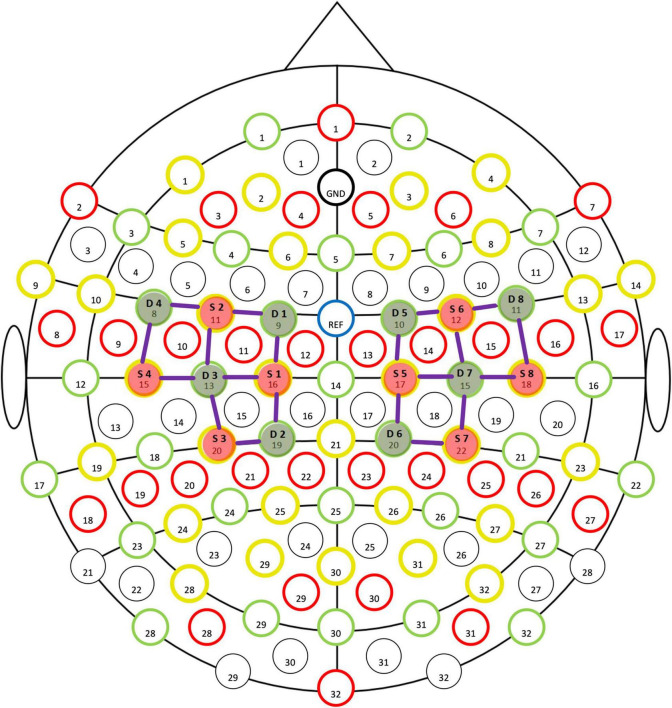
Schematic diagram of placement at the sources (S1–S8) and detector (D1–D8).

Functional near-infrared spectroscopy is a tool that holds particular promise for exercise-related protocols (26), and facilitates the study of cerebral mechanisms with an acceptable degree of spatial and temporal resolution (7). A previous study demonstrated that Oxy-Hb concentration, as tested by fNIRS, was a highly sensitive indicator of any changes in local cerebral blood flow (27). Thus, fNIRS (NIRSport2, NIRX, US) was used in this study to test the children’s concentration of Oxy-Hb in the Primary Motor Cortex (M1) during the task, at a sampling rate of 7.8 Hz. An optical cap was fixed on the head of the child, and movement was set by the system adopting the standard of the “10–20 International Standard lead System” (28) to place sources (S1–S8) and detectors (D1–D8) (Figure 1). The test required the child to complete a 10 m natural straight-line walk and cross a total of 5 obstacles with obstacles placed at 2nd, 4th, 6th, 8th, and 10th m (endpoint). The height of the obstacles were 20 cm. Data was preprocessed by the nirsLAB software through a conversion of raw intensity and optical density data into hemoglobin concentration, using the modified Beer–Lambert law (7).

#### Balance Function Test

The TecnoBody balance system (ProKin 252, TecnoBody, Italy) was adopted, to allow the child to stand naturally in the effective area of the balance table (29). Static balance data was collected for 30 s with eyes open and 30 s with eyes closed, before being processed by the ProKin 3.0 Manual software.

#### Plantar Pressure Distribution Test

The static and dynamic plane pressure distribution was collected using the Freemed plane pressure system (Sensor Medica s.a.s, Roma, Italy) at a frequency of 100 Hz (30). To conduct the static plantar pressure distribution test, the child was required to stand naturally on the effective area of the plantar pressure plate for 5 s, and wait for the completion of the automatic data collection. For the dynamic plantar pressure distribution test, a complete gait cycle was collected for the child when walking continuously for 3 m to the effective area of the plantar pressure plate. Three completed gait cycles were collected for the pre-test and for the post-test, with the resulting data processed by the FreeStep software.

#### 3D Gait Test

The Vicon motion capture system (Oxford Metrics Limited, Oxfordshire, United Kingdom) was used to test the 3D gait of the child (31, 32), at an acquisition frequency of 100 Hz. A total of 16 markers were fixed to the lower limbs of the child, including 2 markers at the left and right sides of the pelvis, and 1 marker at the left and right side of the knee joint, thigh, calf, ankle joint, toe, and heel. The 3D gait test and plantar pressure distribution test were performed simultaneously, where the child was required to walk 3 m without stopping, in the range covered by the 6 cameras used. Data from the three completed gaits made by the child on the effective area of the plantar pressure plate was collected and then processed using the Vicon Nexus 2.6 software.

#### Statistical Analysis

The SPSS software (version 26.0) was used to analyze the aforementioned data. A paired *T*-test was carried out to assess the differences between pre- and post-tests for all neuro-biomechanical parameters. The level of difference significance was set as *P* < 0.05. All data was produced as mean ± standard deviation (SD). For the data tested by fNIRS, the nirsLAB software was used to plot topographic images of Oxy-Hb concentration from the pre- and post-test. The data was then aligned to show color blocks at the corresponding electrode positions when there was a significant difference (*P* < 0.05). If there was no color block, then there was no significant difference presented (*P* > 0.05).

## Results

### Functional Near-Infrared Spectroscopy Test

**FIGURE 2:**
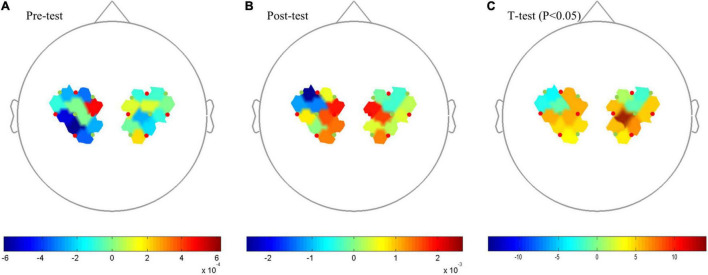
Topography on the Beta image of Oxy-Hb concentration. **(A)** Pre-test Oxy-Hb concentration. **(B)** Post-test of Oxy-Hb concentration. **(C)** *T*-test, *P* < 0.05. Red points indicate sources and green points indicates detectors of the fNIRS system; When the color bar moves from 0 to 10, the larger number (red color) shows that activation became stronger (*P* < 0.05).

The comparison of the Beta values in response to Oxy-Hb concentration between pre-test and post-test (Figures 2A,B) showed that brain activation in Primary Motor Cortex (M1) was significantly increased after 12 weeks of training (*P* < 0.05), specifically in the sources and detectors of S1–D1 (Pre- vs. Post-: 4.76E-04 vs. 1.88E-03), S1–D2 (−2.70E-04 vs. 1.41E-03), S1–D3 (−3.45E-06 vs. 1.46E-03), S2–D1 (−3.31E-04 vs. 4.25E-04), S3–D2 (−3.64E-04 vs. 1.34E-03), S3–D3 (−6.19E-04 vs. 1.30E-04), S4–D3 (−5.20E-04 vs. 7.13E-04), S5–D5 (3.48E-04 vs. 4.88E-04), S5–D6 (−2.90E-05 vs. 4.12E-04), S5–D7 (−2.57E-04 vs. 1.58E-03), S7–D6 (1.96E-04 vs. 1.32E-03), S7–D7 (−2.10E-04 vs. 2.68E-04), S8–D7 (−1.21E-04 vs. 3.89E-04), and S8–D8 (−1.49E-05 vs. 2.42E-04) (Figure 2C).

### Balance Function Test

As seen in Table 2, the static balance parameters comparison between pre-test and post-test showed that the area of motion ellipse and movement length of the child with eyes open decreased significantly (*P* < 0.05), and with eyes closed, area of motion ellipse, back and forth swing, left and right swing and movement length of the child all decreased significantly (*P* < 0.05) after training.

**TABLE 2:** Pre-test and post-test of static balance parameters (unit: mm).

	Pre-test		Post-test	
		
	Open eyes	Close eyes	Open eyes	Close eyes
Area of motion ellipse	135.0 ± 9.6	296.7 ± 17.6	107.3 ± 5.3*	256.0 ± 6.1*
Back and forth swing	6.0 ± 0.8	7.7 ± 0.5	4.0 ± 0.8	5.3 ± 0.5*
Left and right swing	4.0 ± 0.8	5.7 ± 0.9	3.0 ± 0.8	4.0 ± 0.8*
Movement length	318.7 ± 8.6	439.0 ± 14.0	293.7 ± 6.1*	397.3 ± 6.9*

*Significant difference was found between the pre- and post-testing (P < 0.05).

### Plantar Pressure Distribution

**FIGURE 3:**
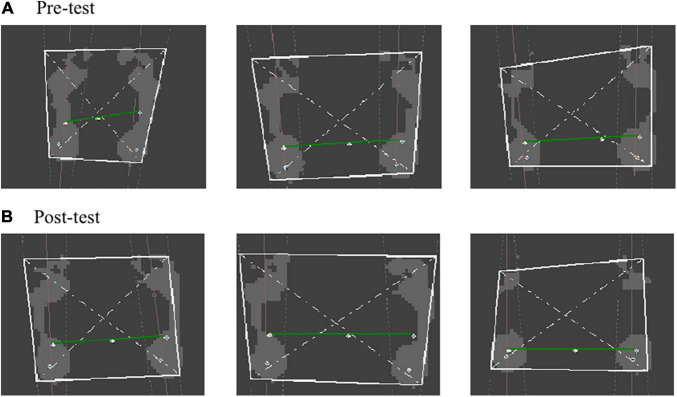
Connecting diagram of static plantar pressure center. **(A)** Pre-test of connecting diagram of static plantar pressure center. **(B)** Post-test of connecting diagram of static plantar pressure center.

The FreeStep plantar pressure distribution system automatically divides the foot into three zones: the forefoot, the midfoot and the rear foot. A comparison of the pre-test and post-test results of the static plantar pressure distribution demonstrates that the pressure center of the left and right foot decreased significantly (*P* < 0.05) from 5.3° dislocation in a straight line in the sagittal plane to 1° (Figure 3). Seen in Table 3, the pre-test and post-test results of dynamic planter pressure distribution showed that both the area size and loading significantly increased in the left foot’s T2-5 and MFL (*P* < 0.05). The area size significantly decreased in the left foot and right foot’s MH1 (*P* < 0.05), but its load significantly increased (*P* < 0.05). Notably, the area size of the left foot’s T1 and MH2-3 decreased (*P* < 0.05). In contrast, the area size significantly increased for MH4-5 of the right foot (*P* < 0.05). In general, an increment of the pressure loading was found on the forefoot of both feet compared with what was recorded seen in the pre-test.

**TABLE 3:** Pre-test and post-test of dynamic plantar pressure parameters.

	Pre-test				Post-test			
		
	Area (cm^2^)		Load (%)		Area (cm^2^)		Load (%)	
				
	Left foot	Right foot	Left foot	Right foot	Left foot	Right foot	Left foot	Right foot
Toe 1 (T1)	4.3 ± 1.0	4.7 ± 0.9	9.3 ± 0.4	13.3 ± 0.9	2.9 ± 0.7*	4.3 ± 0.9	11.3 ± 0.6	17.1 ± 0.9
Toe 2–5 (T2–5)	1.7 ± 0.6	1.3 ± 0.4	3.5 ± 0.6	2.4 ± 0.3	4.5 ± 0.4*	1.9 ± 0.3	6.8 ± 0.3*	3.1 ± 0.1
Metatarsal 1 (MH1)	3.6 ± 0.8	0.7 ± 0.6	7.9 ± 0.8	1.1 ± 0.9	1.7 ± 0.6*	2.2 ± 0.4*	4.1 ± 0.9*	4.7 ± 0.8*
Metatarsal 2–3 (MH2-3)	7.2 ± 1.0	5.1 ± 1.0	16.5 ± 0.5	15.3 ± 0.9	4.3 ± 0.5*	6.4 ± 0.7	15.2 ± 0.7	15.1 ± 0.7
Metatarsal 4–5 (MH4-5)	5.0 ± 1.2	4.6 ± 1.2	11.9 ± 1.9	16.2 ± 1.6	4.6 ± 0.4	10.8 ± 0.9*	12.0 ± 0.4	14.6 ± 0.8
Inside of Arch (MFM)	0.3 ± 0.5	0.6 ± 0.4	0.8 ± 1.1	0.8 ± 0.6	0	0.3 ± 0.5	0	0.2 ± 0.3
Arches of Lateral (MFL)	0.3 ± 0.5	0.3 ± 0.5	0.4 ± 0.5	0.4 ± 0.5	2.4 ± 0.3*	0.3 ± 0.5	3.1 ± 0.2*	0.3 ± 0.4
Inside of Heel (RFM)	7.3 ± 0.9	8.0 ± 1.2	21.6 ± 1.1	27.2 ± 2.2	9.7 ± 0.6	8.3 ± 0.5	21.6 ± 0.4	23.7 ± 0.8
Heel Lateral (RFL)	10.0 ± 1.1	6.1 ± 1.1	28.1 ± 1.2	23.3 ± 0.9	10.9 ± 0.3	7.7 ± 0.9	25.9 ± 0.3	21.2 ± 0.7

*Significant difference was found between the pre- and post-testing (P < 0.05).

### 3D Gait Test

Step width refers to the width of the banner between the midpoint of the heel during the dual support period. Foot axis angle refers to the intersection angle between the line of heel and the second phalange, as well as the included angle of walking direction. The child participating in this test was pigeon-toed, and a comparison of pre-test and post-test gait spatiotemporal parameters (seen in Table 4) showed that the child’s unilateral support phase of the affected leg (left leg) and the non-affected leg (right leg) decreased. On the other hand, the dual support phase shortened significantly (*P* < 0.05). After 12 weeks of training, the swing phase increased significantly (*P* < 0.05) for the affected leg, while the swing phase of the non-affected leg increased more than the affected leg. Step length and step speed increased, while step width and step frequency decreased significantly (*P* < 0.05). The left and right foot axial angles increased significantly (*P* < 0.05) by 2.3° and 2.6°, respectively.

**TABLE 4:** Pre-test and post-test of spatial and temporal parameters of gait and foot axis angle.

	Pre-test		Post-test	
		
	Left leg	Right leg	Left leg	Right leg
Unilateral support phrase (%)	64.8 ± 1.2	74.1 ± 0.7	61.2 ± 0.8*	66.4 ± 0.6*
Swing phrase (%)	35.2 ± 1.2	25.9 ± 0.7	38.8 ± 0.8*	33.6 ± 0.6*
Dual support phase (%)	26.5 ± 0.7		21.8 ± 0.4*	
Step width (cm)	36.3 ± 2.1	29.3 ± 1.7	43.7 ± 1.2*	35.7 ± 1.7*
Stride speed (cm/s)	63.3 ± 4.7		83.3 ± 4.7*	
Stride width (cm)	29.3 ± 2.1		20.3 ± 1.7*	
Stride frequency (step/min)	147.3 ± 2.5		124.3 ± 2.1*	
Foot shaft angle (°)	−5.7 ± 0.7	−1.8 ± 0.7	−3.4 ± 0.3*	0.8 ± 0.4*

*Significant difference was found between the pre- and post-testing (P < 0.05).

The comparison of lower limb kinematics parameters between pre-test and post-test (Table 5) showed that at the moment of left foot landing (t1), the left knee angle increased and the left ankle angle decreased significantly (*P* < 0.05); both the right knee and ankle angles increased significantly at t1 (*P* < 0.05). The left knee angle, right hip, and ankle angles increased significantly (*P* < 0.05) at the right toe off-ground moment (t2). At the moment of right foot landing (t3), the right hip and ankle angles decreased, but the left hip and ankle angles, and both knee angles, increased significantly (*P* < 0.05). The left hip angle, left knee angle, and left ankle angle all increased when the left toe was off the ground (t4), but this was not a significant increase.

**TABLE 5:** Pre-test and post-test of kinematics parameters of the lower extremity (unit:°).

Angles	Pre-test				Post-test			
		
	t1	t2	t3	t4	t1	t2	t3	t4
Left hip	172.0 ± 2.2	168.6.±2.6	173.0 ± 1.6	174.0 ± 2.0	165.0 ± 1.9	173.9 ± 1.7	176.0 ± 1.1	176.5 ± 1.0
Left knee	163.4 ± 2.1	153.4 ± 2.6	149.3 ± 2.3	139.1 ± 2.1	173.6 ± 1.0*	168.2 ± 1.2*	170.2 ± 1.1*	142.8 ± 1.4
Left ankle	117.1 ± 3.0	108.9 ± 2.4	88.8 ± 2.3	110.2 ± 2.3	94.5 ± 2.3*	115.1 ± 1.3	107.1 ± 1.5*	112.5 ± 1.6
Right hip	172.3 ± 1.5	173.2 ± 1.3	166.0 ± 1.7	172.5 ± 1.6	177.7 ± 1.0	178.2 ± 0.8*	159.7 ± 1.1*	175.3 ± 1.1
Right knee	164.4 ± 1.2	145.8 ± 1.7	163.9 ± 1.2	164.9 ± 1.7	172.3 ± 0.8*	147.8 ± 1.0	174.5 ± 1.1*	170.4 ± 0.8
Right ankle	97.7 ± 1.9	99.2 ± 2.0	114.2 ± 1.6	109.3 ± 1.8	106.2 ± 1.0*	105.1 ± 1.1*	101.5 ± 0.9*	114.4 ± 1.2

*Significant difference was found between the pre- and post-testing (P < 0.05).

### Post-clinic Examination

(1) Range of Motion: the result was the same as the result recorded at the admission stage;
(2) Muscle strength: MMT results showed that the muscle groups of the left wrist extensor and bilateral ankle dorsal extensor increased from grade 4 to 5-, and the right wrist extensor, bilateral knee flexors, and bilateral hip flexor increased from grade 4 to 5. All other muscle groups remained at the same grade;
(3) Spasm evaluation: MAS results showed that the grade of the bilateral plantar flexion was elevated to 1, while the rest were 0 grade;
(4) GMFM-88: the total percentage of GMFM-88 was increased from 90.2% at the admission stage to 96.4% after 12 weeks of training.

## Discussion

### Brain Activation and Balance Improvement

Prior to exercise training, the fNIRS results showed that the real-time curves of the child’s Oxy-Hb concentration were irregularly fluctuating during walking, and increasing irregularly when she crossed an obstacle. The child also showed intermittently flustered gait and had poor unilateral support, with the body leaning backward and shaking. Additionally, her upper limbs’ backswing was stiff when crossing obstacles.

The initial target of the exercise prescription was to correct flustered gait. Methods used involved a combination of fast actions and slow motion, including striding, walking, fast walking, variable speed walking, rope ladder exercise, emergency stop, turning, moving back, and racing games to correct the flustered gait. In the 2nd week, the method of correcting flustered gait changed from no resistance to low resistance. Exercise difficulty was increased, in order to assist the child to overcome tension more effectively, and get used to different walking conditions. The above exercises achieved positive results, with the child’s flustered gait being significantly reduced when walking forward. The results of the above exercises were constantly consolidated in the later comprehensive gait resistance exercises.

The intensity of the arm swing was gradually increased between week 3 and week 6, by adding exercises including the hand-held stick arm swing, hand-held dumbbell arm swing, and elastic rope resistance arm swing. The exercises used also included the practice of limiting the width of the swing arms, which would increase the need for coordination of the swing motion. Previously, the child used to have a stiff backswing of the upper arm due to tension, and a diagonally forward arm swing when there was no tension. After 6 weeks of training, her arm swing motion had gradually changed into a fore-backward swing with left and right hands alternating. Then, the child began to practice coordinating the upper and lower limbs by becoming familiar with the motor structure of striding over obstacles with left and right arms swinging in turn, and surmounting obstacles. To cross these obstacles, the child had to enhance her balance and muscle strength, as the maintenance of human balance depends on the integration of sensory and motor systems. These are controlled by the central nervous system, which require an interaction of correct sensory input, information processing, and integration by the central nervous system, along with normal muscle tension, and accurate, rapid responses from the skeletal muscle system. Any abnormality in the process can lead to bodily imbalances and falls. Therefore, balance training was conducted from week 6, with the aim of inducing functional dependence in the cerebral cortex to control the affected limb through the repeated practice of stepping with eyes closed, multi-tasking standing on one foot, multi-tasking Swiss ball exercises, resistance training on balance pads, and balance games. Balance exercise training was also aimed at reducing body sway when the child jumped over obstacles, allowing better stability and sufficient support time for the supporting leg. In addition to balance training, alternate unilateral resistance training was conducted from week 6 to increase the strength of limb muscles, especially the gluteus maximus, quadriceps femoris, gastrocnemius, soleus, and small ankle muscles. One more set of training volume had been added to the affected side to enhance unilateral function and, consequently, to increase body balance. After the 12 weeks of training, the child learned to convert her gradually increasing muscle strength into the ability to control body posture and reduce the amount her body leaned back. This means that the child’s area of motion ellipse, back and forth swing, side to side swing and movement length will be reduced significantly compared to pre-balance testing, particularly with the eyes-closed tests. The noticeable progress of the child’s balance can be primarily explained by the novelty of the progressive unilateral resistance training which integrates traditional coordination and balance training, which is in contrast with common reciprocal leg movements during walking. The specified-side’s subcortical or spinal response might be activated during unilateral training, although the central pattern generators that contribute to bilateral activities might be inhibited. This view has been mentioned in another study conducted by Sukal-Moulton et al. (33), where it was shown that a unilateral cycling task showed higher levels of cortical activity than the bilateral cycling activity, because of the increased postural control demands to stabilize the trunk for unilateral efforts, or possibly due to the influence of subcortical or spinal mechanisms.

Following the 12-week training intervention, fNIRS results showed that the real-time curve of Oxy-Hb concentration fluctuated gently and rose to a peak when the child crossed an obstacle. In turn, five fluctuation rules were set, from gentle to valley peak, across the whole post-test when crossing the five obstacles. Time taken for test subjects to complete the walking test was decreased from 18.6 s to 12.2 s, and the fluster gait disappeared. Through training, the child subject showed higher levels of brain activation, as shown in the post-test results, and performed better motor functions. This highlights the fact that the 12-week exercise intervention might have promoted the child’s functional integration through repeated practice and sensory feedback, before gradually forming dynamic stereotyping and conditioned reflex for the action of jumping over obstacles through the conditional repetitive stimulation of the cerebral cortex.

### Plantar Pressure and Gait Changes

The plantar pressure distribution test was used to collect mechanical parameters of the foot under various posture and movement states. It was also used to reflect the structure and function of the foot and the control status of body posture (34). This would be a critical component when it comes to the provision of medical diagnoses, as well as the completion of biomechanical and rehabilitation research. In this study, the misalignment angle of the plantar pressure test had decreased from 5.3° in the pre-test to only 1° after training in the sagittal plane, which demonstrates that the position of the pressure center dislocated at first and then relocated into the center of the two feet. In addition, standing balance was seen to have significantly increased. The child had learned to coordinate the simultaneous exertion of both feet at the same time. These significant improvements might be due to the balance training which started in week 6, which was mainly aimed at adjusting body posture and center of gravity position. Another reason for these results might be the additional assistance training volume given to the affected (left) foot, as this could have boosted the left foot’s motion sensation and consequently enhanced body balance in the standing position.

The foot’s area of contact with the ground (hereinafter referred to as “area”) reflects the contact area between the foot and the ground, along with plantar pressure distribution. The post-test results of dynamic plantar pressure distribution showed that the contact area characteristics of both feet became like those of normal school-age children (35). The increased stress area could partially relieve damage to local plantar tissues under high pressure initially caused by abnormal walking posture (36). In turn, this could boost walking stability. For a normal child, walking gait becomes stable after the age of 31 months (37) and manifests itself as the gradual transition from the initial ground-touch of rear foot to full-foot landing and then forefoot. This way, the movement process of the stance phase is completed from dorsiflexion (when the rear foot touches the ground) to plantar-flexion (when the forefoot leaves the ground). This is conducive to buffering the impact resistance when landing on the ground and increasing the strength of the pedal when leaving the ground (38). In this study, results relating to dynamic plantar pressure distribution in the pre-test phases showed that both feet had higher posterior load than anterior load, indicating that the child has an abnormal landing position and limited pedal extension compared with a normal child’s gait. Following this, the exercise prescription intervention attached great importance to the correction of forefoot landing to rear foot landing. This emphasized the shift of the body’s center of gravity along with the foot landing, and consolidation of rear foot landing mode in the later comprehensive gait exercise. After 12 weeks of training, an increment of the pressure loading was found on the forefoot of both feet compared with the pre-test. This shows that the load of the child’s left foot gradually shifted from the posterior to the anterior area, and that the child has an improved gait process with dorsal extension when their rear foot was reaching the ground, and has plantar flexion when the forefoot leaves the ground.

Ameliorating gait, as one of the keys to alleviating motor dysfunction, suggests the improvement of neuromuscular control ability and gross motor function. This also has a positive effect on the psychology of children, as it enhances their confidence in the rehabilitation process. In this study, the child had an asymmetrical gait before intervention. The testing results of the 3D gait system showed that she had a shortened time of unilateral support phase and prolonged swing phase on the affected leg, compared to that of the non-affected leg. Furthermore, the dual support phase had also been prolonged. The appearance of this gait feature might be due to the high muscle tension and decreased load-bearing capacity of the affected leg, which would weaken the control ability of the limbs and body balance. Therefore, to keep balance and stability during walking and to avoid falling when walking forward, the child needed to transfer the body’s center of gravity to the non-affected leg as soon as possible when supported by the affected leg. The increased dependence on the non-affected leg as well as prolonged dual support by both legs increased energy consumption during walking, and exacerbated gait abnormalities (39). Thus, one of the most important targets for exercise prescription was to change the child’s asymmetric gait with a shorter dual support phase and a longer swing phase. This exercise prescription gradually improved the child’s gait during the 10 weeks of whole-body resistance exercise (Week 3–12), 4 weeks of coordination-resistance exercise (Week 3–6), and 6 weeks of comprehensive gait-resistance exercise including balance, gait, and resistance exercise (Week 7–12). In contrast to the pre-test findings, post-test results showed a decrease in either unilateral support phase (on both the affected and non-affected legs) or dual support phase, along with an increased swing phase by 7.7% on the affected leg. This denotes that the time asymmetry of gait, and walking ability therein, was greatly ameliorated following the 12 weeks of training. The more compromised human walking stability is, the larger the step width needed to increase the support area between the feet and the ground to improve stability. At the beginning of the exercise prescription intervention, the child’s too-wide step width was a prominent problem that was cause for concern. The child also had a smaller stride length and a slower gait speed than that of normal children (40), which also indicated that the child had weak lower limb strength, walking ability, and balance. In addition, the step frequency of the child (95–125 steps per minute) was significantly higher than that of normal children, and even higher than that of normal adults. The child had an obvious panic gait showing an excessive step frequency and small step length. Hence, the whole-body resistance exercise plan from week 3 focused on the thigh adductor, and in weeks 4–6, training was devoted to correcting step width and flustered gait. The exercises adopted included 10 m double straight-line walking, with a limited width of 25 cm. From week 7 to week 12, the child mainly practiced integrated gait using comprehensive gait exercises, in which the practice of 10 m single straight-line fast walking and walking beyond the body gravity line was adopted, with a limited width of 15 cm. In the last 2 weeks, the child was required to practice powerwalking and variable-velocity walking on a treadmill with a limited width of 15 cm. Following the 12 weeks of training, the child had a 9 cm smaller step width than before training, which suggested positive effects toward gait adjustment. After the comprehensive gait exercises, flustered gait rarely appeared when the child walked forward, and only occasionally appeared in the racing game or when turning. Moreover, the child’s step frequency decreased, while step length and step speed increased in the post-test.

When normal people walk, the lower limbs’ hip, knee, and ankle joints will swing alternately, and cooperate and coordinate with each other continuously. In this study, the left hip angle of the child was 172.0 ± 2.2° when the left foot (affected side) landed on the ground, and the torso was tilted backward without the center of gravity shifting forward. The left knee angle was 163.4 ± 2.1° suggesting an insufficient extension of the left knee. The left ankle angle was 117.1 ± 3.0°, which was significantly greater than that of normal children, indicating that the child landed on the forefoot first with no dorsiflexion. The child was slightly pigeon-toed with both feet bending toward each other when standing and walking, which led to unstable support from the ankle, knee, and hip joints on the affected side. The right-side lower limbs (non-affected side) had better performance than those of the left side when the right foot landed. However, compared with normal children, this child still had problems of larger right hip angle, smaller right knee angle, and larger right ankle angle. As a result, the exercise intervention was mainly carried out using balance exercises and comprehensive gait exercises to transfer the body’s center of gravity, to allow the child’s trunk to lean back no longer during walking, therefore reducing the hip angle at the moment her foot landed. Through lower limb resistance exercise, resistance exercise for ankle joint, forefoot landing gait, correction of “pigeon-toes,” and comprehensive gait exercise were the main focal points. To correct the pigeon-toed landing, a resistance band with a target resistance of 15–30 pounds was used to practice ankle rotation, ankle pump exercise, and heel raising practice with 0.5–2 kg weights to allow toe extension to be practiced, while the ankle support and control ability during walking would also benefit. In the comprehensive gait exercise plan, linear walking and other exercises were adopted. After the intervention, the knee angle of each body side increased at the time of foot landing, while the ankle angle decreased. Forefoot landing was corrected and changed to rear foot landing, especially for the left foot, and dorsiflexion became apparent. The axial angle of the left foot increased, and the pigeon-toed problem was alleviated effectively. The axial angle of the child’s right foot had changed from negative to positive and no pigeon-toed issue was demonstrated. Moreover, the landing mode and overall stability of their gait were significantly improved.

## Conclusion

This study has introduced a personalized exercise prescription designed for the specific situation of a child with VES. The training was adjusted periodically depending on the test subject’s recovery process and response. 12 weeks of individualized exercise training was shown to potentially enhance activation of motor areas and improve gait and balance function. The results suggest that exercise prescription can be used as a viable method of improvement for the rehabilitation and recovery of motor function in a child with VES. The neuro-biomechanics testing used in this study can be used to evaluate the child’s brain activation and motor function responses as well. Crucially, the results presented can provide a reference for subsequent clinical studies on children with VES, but a larger sample size is needed to determine the suitability of these methods for similar cases.

## Data Availability Statement

The original contributions presented in the study are included in the article/supplementary material, further inquiries can be directed to the corresponding author.

## Ethics Statement

The studies involving human participants were reviewed and approved by Medical Ethics Committee Sichuan Bayi Rehabilitation Center (Sichuan Province Rehabilitation Hospital). Written informed consent to participate in this study was provided by the participants’ legal guardian/next of kin. Written informed consent was obtained from the minor(s)’ legal guardian/next of kin for the publication of any potentially identifiable images or data included in this article.

## Author Contributions

YW, XK, and JZ : study conception, design, and methodology. XK and JZ: tool and supervision. YW and LZ: exercise prescription procedure and data curation. YW, JJ, and JZ: writing–original draft preparation. YW, JJ, XK, BCC, JB, and SL: writing–review and editing. All authors contributed to manuscript revision, read, and approved the submitted version.

## Conflict of Interest

The authors declare that the research was conducted in the absence of any commercial or financial relationships that could be construed as a potential conflict of interest.

## Publisher’s Note

All claims expressed in this article are solely those of the authors and do not necessarily represent those of their affiliated organizations, or those of the publisher, the editors and the reviewers. Any product that may be evaluated in this article, or claim that may be made by its manufacturer, is not guaranteed or endorsed by the publisher.
